# Extraction of Foreign Bodies from the Ear by Syringing with Cold Water

**Published:** 1842-05

**Authors:** 


*Extraction of Foreign Bodies from the Ear by Syringing with cold water.*—Mr. Carpenter of Castlecomer has successfully employed injections of cold water for the removal of foreign bodies from the ear. "The first case," he observes, "brought to me, some years back, was one in which the foreign body was a garden pea, which, as is usual, was pushed in as far as the tympanum by the interference of the child's friends. The instrument I selected was a very small forceps with blunt points, with which I could catch the pea, but could not move it, and, on endeavoring to do so, caused intolerable pain. It immediately occurred to me to inject cold water with force, in order to at least displace the pea, and perhaps thereby render it more manageable—I did so with a two-ounce syringe, and found the pea so far displaced as to lie at the orifice of the meatus, whence I removed it without further trouble or pain to the child. Since that I have extracted many peas in the same way—in some instances they have lain at the orifice, and in others been forced out completely. So far I was satisfied that peas could be removed by the above method, and will admit that I felt anxious to have an opportunity of trying my hand on something of more difficult extraction. About six months back that offered—the substance was a pebble of a most irregular shape, and so large that I was surprised how the child could have borne to introduce it—the meatus was not inflamed; but the pebble was so far in, and so wedged in its place, that to touch it ever so lightly with any instrument, was almost enough to throw the child into convulsions. I almost despaired of succeeding with the syringe, but made the trial, and not having a two ounce one at hand, used a pint one—the first injection failed; but, while using a second, the pebble, much to my satisfaction, was forced out on the napkin held under.

In the last case brought to me I could hardly feel the foreign body with a silver probe, nor could the child's parents tell me what it was; however, knowing it to be small, I used a small sized syringe, and by one injection had a grain of oats at the orifice of the meatus.—*Dublin Medical Press*, June 20th, 1841.

